# Lung and Nodal Involvement in Nontuberculous Mycobacterial Disease: PET/CT Role

**DOI:** 10.1155/2015/353202

**Published:** 2015-05-28

**Authors:** Ginevra Del Giudice, Andrea Bianco, Antonio Cennamo, Giulia Santoro, Marco Bifulco, Carlo Marzo, Gennaro Mazzarella

**Affiliations:** ^1^Department of Cardiothoracic and Respiratory Sciences, Second University of Naples, “dei Colli” Hospital, Via Leonardo Bianchi, 80131 Naples, Italy; ^2^Department of Medicine and Health Sciences, University of Molise, Via Giovanni Paolo II, Loc. Tappino, 86100 Campobasso, Italy; ^3^Department of Laboratory Pathology and Microbiology, Section of Mycobacteriology, “dei Colli” Hospital, Via Leonardo Bianchi, 80131 Naples, Italy; ^4^Department of Radiology, Section of Nuclear Medicine, “dei Colli” Hospital, Via Leonardo Bianchi, 80131 Naples, Italy

## Abstract

*Introduction*. Systematic use of ^18^F-FDG PET/CT has the potential to simultaneously assess both pulmonary and lymph node involvement in nontuberculous mycobacterial (NTM) lung infection. *Objective*. The aim of the study was to evaluate the role of ^18^F-FDG PET/CT in the assessment of both mediastinal lymph nodes and lung involvement in NTM patients compared with active tuberculosis (TB) patients. *Methods*. 26 patients with pulmonary NTM disease were selected; six consecutive patients had undergone ^18^F-FDG PET/CT and data was compared with 6 active TB patients. *Results*. NTM exhibited different radiological lung patterns with an average SUV max value at PET/CT scan of 3,59 ± 2,32 (range 1,14 to 9,01) on pulmonary lesions and a mean value of SUV max 1,21 ± 0,29 (range 0,90 to 1,70) on mediastinal lymph nodes. Pulmonary lesions in TB showed an average SUV max value of 10,07 ± 6,45 (range 1,20 to 22,75) whilst involved mediastinal lymph nodes exhibited a mean SUV max value of 7,23 ± 3,03 (range 1,78 to 15,72). *Conclusions*. The differences in PET uptake in a broad range of lung lesions and lymph nodes between NTM and *M. tuberculosis* patients suggest a potential role for PET/CT scan in the diagnosis and management of pulmonary mycobacterial disease.

## 1. Introduction

Epidemiological observations indicate an increase in the prevalence of NTM disease worldwide.

NTM infection is caused by a group of versatile opportunistic bacterial pathogens and remains underdiagnosed due to difficulties in pathogen isolation and nonspecificity of clinical signs. Isolation of NTM from respiratory specimens does not always indicate disease; clinical, radiographic, and microbiologic criteria must all be met to make a diagnosis of NTM lung disease according to ATS guidelines [1, 2].

Although ATS criteria represent the cornerstone of NTM diagnosis, some limitations need to be addressed: the uncertain significance of isolation of NTM; the experience and training required in using specific microbiological diagnostic kits; and use of X-ray investigations for NTM identification.

Radiological patterns of lung NTM disease include nodular lesions, cavitary pattern, bronchiectasis, lobar consolidation, and consolidation associated with fibrothorax, as well as possible mix patterns (e.g., nodular-bronchiectasis) [3]. Lymphadenitis is the main clinical manifestation of NTM in children aged 1–5 years [4, 5], while skin and soft tissue involvement is not uncommon.

Lymph node involvement in NTM is not easily interpreted at thoracic CT scan unlike* M. tuberculosis* lung infections; NTM patients do not have a primary complex, colliquation of nodal stations is not detectable, and lymph node repair in calcification or fibrocalcification is absent.

^18^F-FDG positron emission tomography-computed tomography (PET/CT) is a functional imaging technique which detects metabolically active areas through the accumulation of the radiotracer 18-fluorodeoxyglucose. PET/CT is widely used in cancer management [6, 7] and is already employed in the assessment of inflammatory/infectious respiratory diseases with lymph node involvement such as sarcoidosis, TB, and intracellular infections [8–11].

The purpose of the study was to assess and compare, through the use of ^18^F-FDG PET/CT, lung and mediastinal lymph node involvement in patients with NTM and patients with active TB. The data were collected at “Vincenzo Monaldi” Hospital, Naples, center for Phthisiology and clinical laboratory investigation in mycobacteriology.

## 2. Materials and Methods

Between 2008 and 2011 79 biological culture samples positive for NTM were evaluated in our mycobacteriology center.

In order to make an accurate disease diagnosis, according to ATS 2007 guidelines for NTM lung pathology, clinical, microbiological, and radiological criteria were all considered. 26 NTM patients were enrolled.

During the same period (2008–2011) 486 samples positive for* Mycobacterium tuberculosis* were evaluated. Only 6 patients affected by active TB according to medical history, clinical, radiology, and acute-phase reactants underwent ^18^F-FDG PET/CT.

The clinical records and radiological examinations of patients with positive cultures for NTM species and TB by sputum or bronchial wash were reviewed retrospectively, including information about age, sex, respiratory symptoms, preexistent pulmonary and nonpulmonary illnesses, results of anti-HIV antibody, number of positive isolates, and available lung biopsy results (Table 1).

Six patients with NTM infection and 6 patients with diagnosis of TB who underwent an ^18^F-FDG PET/CT were considered eligible for the study.

### 2.1. NTM Patients

All were male, Caucasian, with mean age of 61 (between 38 and 77 years), HIV negative presenting with nonspecific symptoms such as cough with sputum, evening fever, and dyspnea. Comorbidities such as COPD, diabetes mellitus, and bronchial and allergic asthma were present, and one patient had received an aortic valve replacement. Four NTM patients had a positive microbiological diagnosis on sputum samples (*M. kansasii n* = 2,* MAC n* = 1, and* M. xenopi n* = 1) and two on bronchial aspirate (*M. kansasii n* = 1,* MAC n* = 1).

### 2.2. Active Tuberculosis Patients

Three females and three males, Caucasian, were selected, with mean age of 52 (between 34 and 78 years). Symptoms included fever, dry tickly cough with or without sputum, weight loss, and episodes of hemoptysis; C reactive protein (PCR) and sedimentation rate were mostly raised. Four patients presented with comorbidities such as diabetes mellitus, sinus tachycardia, HBV+, atrial fibrillation, arterial hypertension, hemorrhoids, rheumatoid arthritis, and ischemic heart disease.* M. tuberculosis* isolation in five patients was made on bronchial aspirate and in one on sputum.

### 2.3. Microbiological Technique

Microscopic examinations were made with Ziehl-Neelsen coloration and confirmed on samples of sputum or bronchial aspirate using trough techniques of gene amplification (PCR), cultures on solid media (Lowenstein-Jensen) and liquid, and radiometric technique BACTEC MGIT 960. DNA probes were performed for molecular speciation: the technique of gene amplification PCR, and the technique INNO-LiPA mycobacterium V2.

### 2.4. ^18^F-FDG PET/CT Imaging

Patients underwent ^18^F-FDG PET/CT using a whole-body scanner (Siemens Biograph 16 PET-CT scanner). Patients were fasted prior to scanning for at least 4–6 hours, well hydrated, with the recommendation not to undergo intense physical activity the day before and blood glucose levels were assessed to ensure values <150 mg/dL. For diabetic patients insulin was administered three hours before to normalize blood glucose level. After establishment of venous access, the dose of radiotracer (^18^F-FDG) was administered according to the weight of the patient (5.18 MBq/kg). Expected average dose is 370–555 MBq. The administration of the radiotracer was followed by oral hydration (to promote an appropriate distribution of the tracer in the tissues and its urinary excretion) and by a rest period in which the patient was informed not to walk, speak, or make any kind of effort to avoid the physiological uptake by muscle overactivity especially at the level of the eye muscles and vocal cords. 50 minutes after the injection of ^18^F-FDG the patient was positioned supine in the PET scanner. The PET/CT was composed of a dedicated PET scanner with a detector LSO with crystal dimensions of 4.0 × 4.0 × 20 mm, transaxial field of view of 585 mm, an axial field of view of 162 mm, intrinsic axial and transaxial resolution that was between 4.6 and 5.8 mm, and a multislice CT. The parameters used for the acquisition included CT 120 kV, 80 mA, 16 slices helical, and 0.5 s for rotation. Initial TC without contrast was carried out followed by a PET scan performed on the body of the patient in the same position from the head to knee level. In order to better assess the involvement of the lung and lymph node.

Initial CT without contrast was carried out followed by a PET scan performed on the body of the patient in the same position from the head to knee level.

After reconstruction of the coronal, sagittal, and transverse planes, the images were interpreted qualitatively and semiquantitatively with the standardized uptake value (SUV).

An experienced radiologist in nuclear medicine evaluated radiotracer uptake in mediastinal lymph nodes.

### 2.5. Statistical Analysis

The values were expressed as mean ± SD and were compared using the Mann-Whitney *U* test for two unpaired samples. Differences were considered statistically significant when *P* < 0,05. The results have been obtained through the use of MATLAB program.

## 3. Results

**Figure 1:**
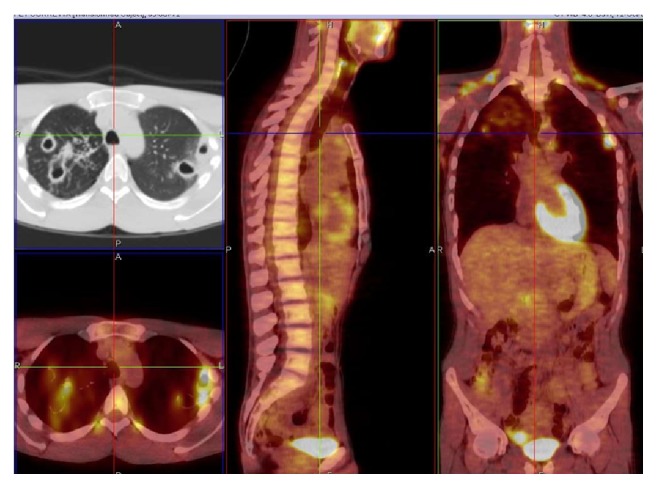
*M. kansasii* infection, cavitary lesions in the right upper lobe and in the apical segment of the right lower lobe and left upper lobe, SUV max 5.36. Related to patient 3 (PET/CT. Courtesy “V. Monaldi” Hospital).

NTM patients CT scans showed nodular or pseudonodular involvement (*n* = 3), parenchymal consolidation (*n* = 2), pleural thickening (*n* = 2), calcified sequelae (*n* = 2), cavitary lesions (*n* = 2), and pleural effusion (*n* = 1). More than one radiological pattern was simultaneously found in five NTM patients. PET/CT scan in NTM patients showed areas of consolidation or parenchymal thickening with SUV max between 1,64 and 9,01 (*n* = 3), pleural thickenings with SUV max that ranged from 1,14 to 1,80 (*n* = 2), subpleural nodular lesions with SUV max 2,7–4,5 (*n* = 1), and multiple cavitary lesions with SUV max between 2,60 and 5,36 (*n* = 1) (Figure 1), and in one patient a baseline monolateral pleural effusion with maximum uptake (SUV max of 6,06) was observed.

One patient had no lesions with significant uptake.

The mean value of the SUV max of these metabolically active areas was 3,59 ± 2,32 (range 1,14 to 9,01).

**Figure 2:**
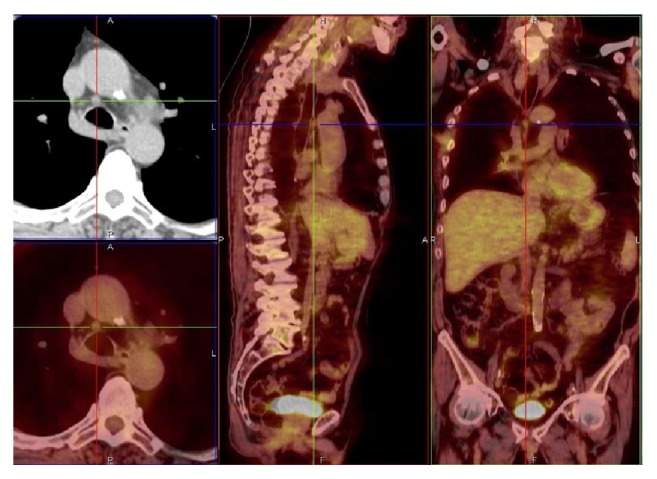
*M. xenopi* infection, lower right paratracheal lymph node (4R), SUV max 1.19. Related to patient 2 (PET/CT. Courtesy “V. Monaldi” Hospital).

**Figure 3:**
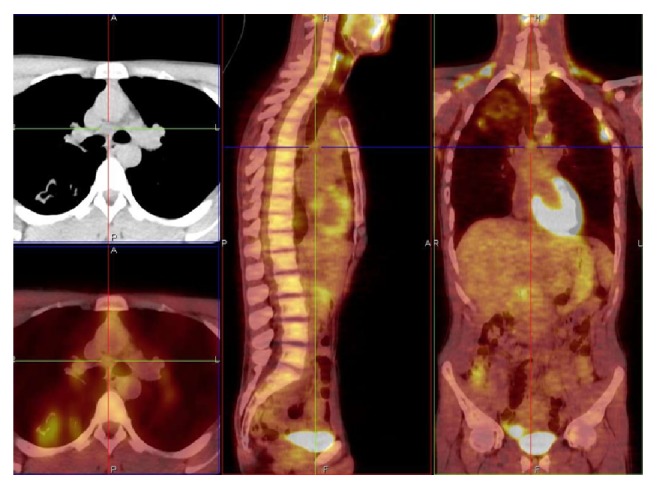
*M. kansasii* infection, subcarinal lymph node (7), SUV max 1.20. Related to patient 3 (PET/CT. Courtesy “V. Monaldi” Hospital).

Carinal, precarinal, paratracheal, and prevascular lymph nodes exhibited an average value of SUV max 1,21 ± 0,29 (range 0,96 to 1,70) (Figures 2 and 3).

Table 2 reports the details of the mediastinal lymph node involvement in NTM patients according to the classification revisited by Mountain.

**Figure 4:**
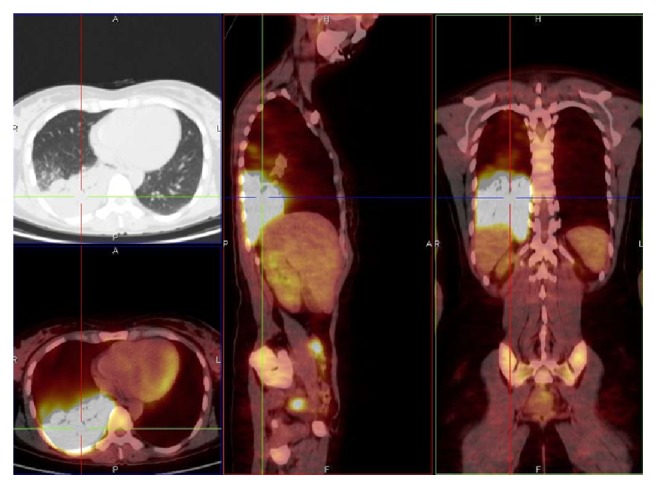
*M. tuberculosis* infection. Massive area of high metabolic activity, SUV max 19.55, lower right lobe. Related to patient 11 (PET/CT. Courtesy “V. Monaldi” Hospital).

At CT scan more than one radiological pattern was simultaneously found in active tuberculosis patients. CT findings included parenchymal consolidation or ground glass opacity (GGO) (*n* = 6) with SUV max between 1,20 and 22,75, excavation (*n* = 3) SUV max 4,19–15,10, and miliary (*n* = 1) SUV max 8,25, with lymph node involvement; in one case a combination of nodular consolidation PET-positive and bilateral pleural parietobasal effusion PET-negative was identified. PET/CT showed the presence (for all of 6 patients) of extensive areas of high metabolic activity (SUV max range 1,20–22,75 (Figure 4) and average values of SUV max of 10,07 ± 6,45).

**Figure 5:**
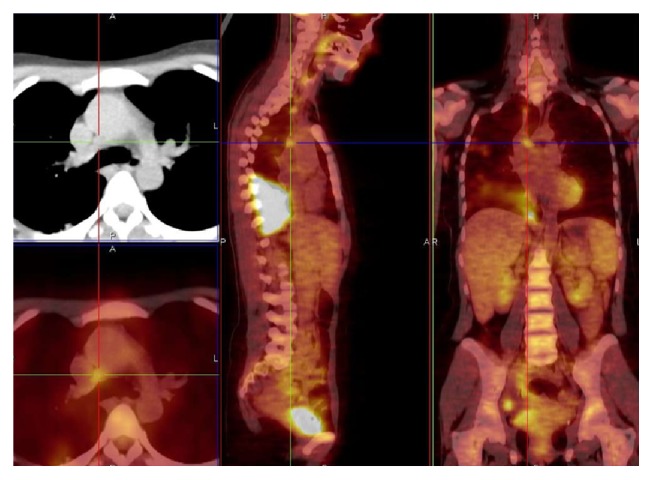
*M. tuberculosis* infection with lymph node of Barety space (4R), SUV max 3.50. Related to patient 11 (PET/CT. Courtesy “V. Monaldi” Hospital).

In addition, 5 patients showed increased metabolic activity in paratracheal lymph nodes, Barety space (Figure 5), prevascular, carinal, paraesophageal, hilar, and aortopulmonary window and axillary with mean values of SUV max of 7,23 ± 3,03 (range 1,78–15,72).

Table 3 reports the details of the mediastinal lymph nodes in active TB.

The data obtained with PET/CT (Table 4) shows that patients with active TB have high metabolic activity in lung lesions and in mediastinal lymph nodes compared to NTM. Furthermore NTM patients, while presenting with lung lesions exhibiting high uptake, showed little or no lymph node involvement.

**Figure 6:**
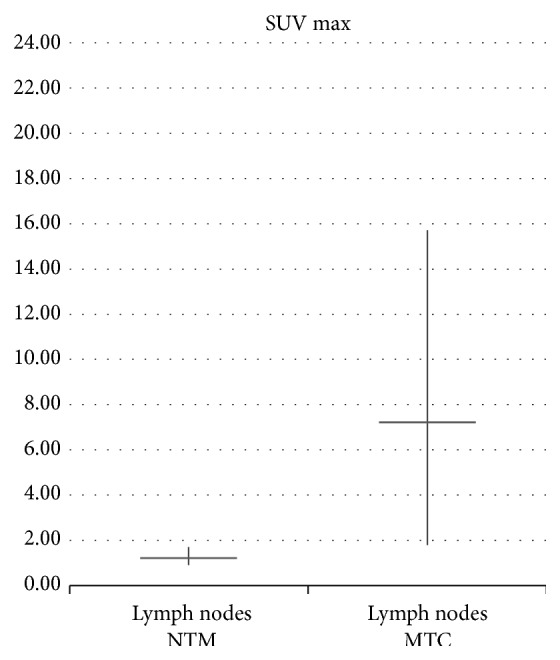
Comparison between average SUV max of mediastinal lymph nodes in NTM and* M. tuberculosis* patients.

**Figure 7:**
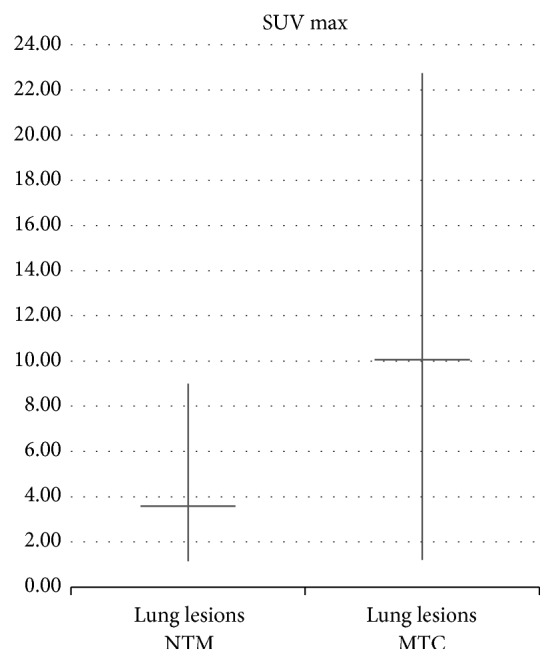
Comparison between average SUV max of lung lesions in NTM and* M. tuberculosis* patients.

Patients with active TB showed a mean value of SUV max of mediastinal lymph nodes equal to 7,23 ± 3,03 (range 1,78 to 15,72), significantly higher than the average value of SUV max 1,21 ± 0,29 (range 0,90 to 1,70); *P* = 2, 62 × 10^−6^ detected in patients with NTM (Figure 6). The average SUV max of lung lesions in patients with active TB was equal to 10,07 ± 6,45 (range 1,20 to 22,75) and significantly higher than the average SUV max of lung lesions in NTM patients 3,59 ± 2,32 (range 1,14 to 9,01); *P* = 0,0043 (Figure 7).

## 4. Discussion

Our study suggests that ^18^F-FDG PET/CT is a useful diagnostic technique which may be helpful in the management of pulmonary NTM and* M. tuberculosis* infections.

Indeed we have demonstrated that both NTM and* M. tuberculosis* patients exhibit an increase of SUV in lung lesions although average values were less in NTM than in* M. tuberculosis*; lymph nodes SUV value was low or zero in NMT whilst results were high in active tuberculosis patients.

Despite well-established diagnostic criteria, NTM lung disease remains challenging for clinicians and new approaches are required for improving disease management.

The literature provides only a few studies regarding the potential usefulness of PET/CT as a diagnostic tool in nontuberculous mycobacteriosis.

Currently the ^18^F-FDG PET/CT is widely used in cancer management for diagnosis, staging, and response to therapy [7, 12]. There is now a growing interest in the potential role of ^18^F-FDG PET in the functional assessment of inflammatory and infectious pulmonary diseases including mycobacteria.

A study conducted by Goo et al. showed that the pulmonary tuberculoma commonly causes an increase in uptake of ^18^F-FDG suggesting caution in differentiating benign from malignant pulmonary abnormalities [13].

Similarly, the literature reports various cases of pulmonary nodular lesions with PET/CT high uptake caused by infection with NTM (especially MAC) whose differential diagnosis with malignant nodule was performed through histological typing [14, 15].

Another study by Demura et al. evaluated the use of ^18^F-FDG PET/CT in the diagnosis and monitoring of therapy for mycobacteriosis. The average uptake was higher in patients with MAC than those with TB. The study also confirms that, following one or two years of treatment for MAC, despite the persistence of nodules on HRCT, the ^18^F-FDG PET uptake was more likely to correlate with disease state [16].

Our data differ from that reported by Demura, which may be due to differing patient selection criteria including the lung lesion characteristics and subtype of NTM species in patients enrolled.

Davis et al. have shown the utility of PET/CT to detect tuberculous lesions and to monitor response to chemotherapy in animal models hypothesizing the possible application for humans [17].

In our study we have demonstrated the usefulness of ^18^F-FDG PET/CT in reflecting the activity and extent of disease by monitoring metabolic activity not only in nodular lesions but also within a broad range of radiologically visible lung lesions in NTM and* M. tuberculosis*. This is the first study in which PET/CT characteristics of lesions different from tuberculoma or single nodular lesion have been evaluated.

The broad range of radiological patterns seen in NTM and* M. tuberculosis* includes nodular or pseudonodular lesions, parenchymal consolidation, cavitary lesions, pleural thickenings, and pleural effusions and may reflect differences in cellular immune response at the level of the diseased lung [18–20].

One of the most important findings from this study is the role of ^18^F-FDG PET/CT in the comparative evaluation of both pulmonary lesions and mediastinal lymph nodes in patients with NTM and those with active TB.

Our results have shown that, in both NTM and* M. tuberculosis*, increased uptake of the radioisotope at PET/CT scan is representative of active inflammatory areas within lung lesions which appears to reflect the real extent of disease.

PET/CT scan allows simultaneous assessment of both parenchymal and lymph node lesions which is of great value in tuberculous disease where lung involvement is almost always accompanied by lymphadenopathy.

Although performed on a small number of patients, our observations suggest a potential role for PET/CT in assessing extent of disease, evolution, and follow-up in both NTM and* M. tuberculosis* patients. Further investigations are required to verify whether there is the possibility to identify specific SUV cutoffs which in turn may define the potential indications and limitations of PET/CT scan in both pulmonary NTM and* M. tuberculosis* infections.

## Figures and Tables

**Table 1:** The clinical records and radiological examination of patients with positive cultures for NTM species and *M. tuberculosis*.

Patient	Age	Sex	Agent isolated	Sample	Anti-HIV antibody	ESR	Clinic	Comorbidity	Lymph node	Lymph node uptake PET (SUV max)	Radiological lung patterns	Lung lesion uptake PET (SUV max)
1	40	M	*M. kansasii *	BAS	—	18	Evening fever	Bronchial and allergic asthma	4 R	1,03	Small consolidation LUL	No uptake
									3	0,92		
									4 L	1,04		

2	77	M	*M. xenopi *	Sputum	—	24	Fever, cough	Diabetes mellitus, arterial hypertension	7	1,68	Pseudonodular consolidation RLL	9,01
									3	1,50		
											Right basal pleural effusion	6,06
									4 R	1,19		

3	38	M	*M. kansasii *	Sputum	—	31	Fever, cough with sputum	No	3	1,70	Cavitary lesion RUL	2,60
											Cavity apical segment RLL	3,15
									7	1,20		
											Cavity LUL	5,36

4	70	M	*MAC *	Sputum	—	21	Evening fever, cough with mucopurulent sputum	COPD, diabetes mellitus	7	0,9	Right posterior basal pleural thickening	1,80
											Left posterior basal pleural thickening	1,14
											Calcification of the anterior segment of RUL	No uptake

5	67	M	*M. kansasii *	Sputum	—	27	Cough, dyspnea	COPD, arterial hypertension	4 L	0,96	Apical nodular consolidation RUL	1,64
											Pleural thickening anterior basal lateral right	1,39

6	76	M	*MAC *	BAS	—	23	Dyspnea on exertion, evening fever	Aortic valve replacement	7	1,16	Subpleural nodular consolidation anterior segment RUL	4,50
											Subpleural nodular consolidations LUL	2,70
											Mild bilateral parenchymal consolidation	3,70
											Mantle calcified sequelae of UL and of left hilum	No uptake

7	34	M	*M. tuberculosis *	BAS	—	94	Fever, cough with sputum	No	2 R	15,72	Consolidation + small cavitary lesions apical segment RUL	15,10
									4 R	6,93		
											Basal consolidation segments RLL	22,75
									10 R	7,55	GGO + micronodules of apical segment LUL	3,60
									7	8,20		

8	49	M	*M. tuberculosis *	BAS	—	63	Fever, cough with sputum	Arterial hypertension	2 R	8,55	Diffuse subpleural consolidation of right lung	11,58
									2 L	8,55		
									3	8,55	Diffuse subpleural consolidation of left lung	12,27
									4 R	8,55		
									4 L	8,55	Consolidation of posterior segment RUL	12,24
									5	8,55		
									7	8,55	Consolidation of apical-dorsal segment LUL	17,06
									8	8,55		
									10 R 10 L	8,55 8,55	Diffuse micronodules in both lungs (miliary)	8,95

9	61	F	*M. tuberculosis *	BAS	—	27	Weight loss, cough with sputum	Diabetes mellitus, sinus tachycardia, HBV+	2 R	3,30	Consolidation with excavation and various contextual calcifications of posterior segment RUL	4,19
									3	8,10		
									4 R	5,90		
									8	7,70		
									10 R	7,56		
									Right axillary	12,94		
									Retromandibular left	7,60		

10	78	F	*M. tuberculosis *	BAS	—	79	Fever, dry cough	Atrial fibrillation, arterial hypertension, and hemorrhoids	No linphonodes		Multiple focal bilateral consolidations; the greater is the left hilar	8,00
											Bilateral pleural effusions	No uptake

11	39	F	*M. tuberculosis *	Sputum	—	61	Fever, dyspnea on exertion	No	2 R	2,77	Consolidation of posterior and lateral basal segments RUL	19,55
									4 R	3,50	Consolidation RUL	3,54

12	51	M	*M. tuberculosis *	BAS	—	32	Hemoptysis, fever	Rheumatoid arthritis, ischemic heart disease	3	1,78	Cavity apical segment LUL	6,55
									5	1,78	Intrascissural left consolidation	4,53
									Left axillary	4,17	Lung consolidation RUL	1,20

GGO: ground glass opacity. RUL: right upper lobe. LUL: left upper lobe. BAS: bronchoaspirate. ESR: erythrocyte sedimentation rate.

**Table 2:** SUV max of mediastinal lymph nodes detected by PET/CT in patients with lung NTM disease.

Patient	Mediastinal lymph nodes	SUV max
1	Station 4 R	1,03
	Station 4 L	1,04
	Station 3	0,92

2	Station 7	1,68
	Station 4 R	1,19
	Station 3	1,50

3	Station 3	1,70
	Station 7	1,20

4	Station 7	0,90

5	Station 4 L	0,96

6	Station 7	1,16

**Table 3:** SUV max of mediastinal lymph nodes detected by PET/CT in patients with TB.

Patient	Mediastinal lymphonodes	SUV max
7	Station 2 R	15,72
	Station 4 R	6,93
	Station 10 R	7,55
	Station 7	8,20

8	Station 2 R	8,55
	Station 2 L	8,55
	Station 3	8,55
	Station 4 R	8,55
	Station 4 L	8,55
	Station 5	8,55
	Station 7	8,55
	Station 8	8,55
	Station 10 R	8,55
	Station 10 L	8,55
	Station 8	8,55
	Station 10 R	8,55
	Station 10 L	8,55

9	Station 2 R	3,30
	Station 3	8,10
	Station 4 R	5,90
	Station 8	7,70
	Station 10 R	7,56

10	No lymph nodes	

11	Station 2 R	2,77
	Station 4 R	3,50

12	Station 3	1,78
	Station 5	1,78

**Table 4:** SUV max of the mediastinal lymph nodes and lung hypermetabolic areas in patients with NTM and TB.

	Mean value SUV max	Range
Mediastinal lymph nodes NTM patients	1,21 ± 0,29	0,90–1,70
Hypermetabolic lung areas NTM patients	3,59 ± 2,32	1,14–9,01
Mediastinal lymph nodes TB patients	7,23 ± 3,03	1,78–15,72
Hypermetabolic lung areas TB patients	10,07 ± 6,45	1,20–22,75

## References

[1] Griffith D. E., Aksamit T., Brown-Elliott B. A. (2007). An official ATS/IDSA statement: diagnosis, treatment, and prevention of nontuberculous mycobacterial diseases. *The American Journal of Respiratory and Critical Care Medicine*.

[2] Griffith D. E., Aksamit T., Brown-Elliott B. A. (2007). An official ATS/IDSA statement: diagnosis, treatment, and prevention of nontuberculous mycobacterial diseases. *The American Journal of Respiratory and Critical Care Medicine*.

[3] del Giudice G., Iadevaia C., Santoro G., Moscariello E., Smeraglia R., Marzo C. (2011). Nontuberculous mycobacterial lung disease in patients without HIV infection: a retrospective analysis over 3 years. *Clinical Respiratory Journal*.

[4] Lincoln E. M., Gilbert L. A. (1972). Disease in children due to mycobacteria other than *Mycobacterium tuberculosis*. *The American Review of Respiratory Disease*.

[5] Hazra R., Robson C. D., Perez-Atayde A. R., Husson R. N. (1999). Lymphadenitis due to nontuberculous mycobacteria in children: presentation and response to therapy. *Clinical Infectious Diseases*.

[6] Schöder H., Gönen M. (2007). Screening for cancer with PET and PET/CT: potential and limitations. *Journal of Nuclear Medicine*.

[7] Brunese L., Greco B., Setola F. R. (2013). Non-small cell lung cancer evaluated with quantitative contrast-enhanced CT and PET-CT: net enhancement and standardized uptake values are related to tumour size and histology. *Medical Science Monitor*.

[8] Teirstein A. S., Machac J., Almeida O., Lu P., Padilla M. L., Iannuzzi M. C. (2007). Results of 188 whole-body fluorodeoxyglucose positron emission tomography scans in 137 patients with sarcoidosis. *Chest*.

[9] Braun J. J., Kessler R., Constantinesco A., Imperiale A. (2008). 18F-FDG PET/CT in sarcoidosis management: Review and report of 20 cases. *European Journal of Nuclear Medicine and Molecular Imaging*.

[10] Schuurmans M. M., Ellmann A., Bouma H., Diacon A. H., Dyckmans K., Bolliger C. T. (2007). Solitary pulmonary nodule evaluation with 99mTc-methoxy isobutyl isonitrile in a tuberculosis-endemic area. *European Respiratory Journal*.

[11] Bianco A., Mazzarella G., Rocco D., Gasperi M., di Marco R., Brunese L. (2010). FDG/PET uptake in asymptomatic multilobar chlamydia pneumoniae pneumonia. *Medical Science Monitor*.

[12] Krause B. J., Schwarzenböck S., Souvatzoglou M. (2013). FDG PET and PET/CT. *Recent Results in Cancer Research*.

[13] Goo J. M., Im J. G., Do K. H. (2000). Pulmonary tuberculoma evaluated by means of FDG PET: findings in 10 cases. *Radiology*.

[14] Bandoh S., Fujita J., Ueda Y. (2003). Uptake of fluorine-18-fluorodeoxyglucose in pulmonary *Mycobacterium avium* complex infection. *Internal Medicine*.

[15] Matsuyama W., Yamamoto M., Oonakahara K.-I. (2004). Intense 18F-fluorodeoxyglucose uptake caused by pulmonary Mycobacterium intracellulare infection. *Nihon Kokyuki Gakkai Zasshi*.

[16] Demura Y., Tsuchida T., Uesaka D. (2009). Usefulness of 18F-fluorodeoxyglucose positron emission tomography for diagnosing disease activity and monitoring therapeutic response in patients with pulmonary mycobacteriosis. *European Journal of Nuclear Medicine and Molecular Imaging*.

[17] Davis S. L., Nuermberger E. L., Um P. K. (2009). Noninvasive pulmonary [^18^F]-2-fluoro-deoxy-D-glucose positron emission tomography correlates with bactericidal activity of tuberculosis drug treatment. *Antimicrobial Agents and Chemotherapy*.

[18] Condos R., Rom W. N., Liu Y. M., Schluger N. W. (1998). Local immune responses correlate with presentation and outcome in tuberculosis. *American Journal of Respiratory and Critical Care Medicine*.

[19] Mazzarella G., Bianco A., Perna F. (2003). T lymphocyte phenotypic profile in lung segments affected by cavitary and non-cavitary tuberculosis. *Clinical and Experimental Immunology*.

[20] Sanduzzi A., Perna F., Galgani M., Bianco A., Mazzarella G. (2005). Lung and peripheral blood T lymphocytes IFN-*γ* production in infliximab-associated pulmonary tuberculosis. *Respiratory Medicine Extra*.

